# Resolution of fibrosis in mdx dystrophic mouse after oral consumption of N-163 strain of *Aureobasidium pullulans* produced β-glucan

**DOI:** 10.1038/s41598-023-44330-0

**Published:** 2023-10-09

**Authors:** Senthilkumar Preethy, Yoshitsugu Aoki, Katsura Minegishi, Masaru Iwasaki, Rajappa Senthilkumar, Samuel J. K. Abraham

**Affiliations:** 1 Fujio-Eiji Academic Terrain (FEAT), Nichi-In Centre for Regenerative Medicine (NCRM), Chennai, India; 2 Department of Molecular Therapy, National Institute of Neuroscience, National Center of Neurology and Psychiatry (NCNP), Tokyo, Japan; 3 II Department of Surgery, Centre for Advancing Clinical Research (CACR), Faculty of Medicine, University of Yamanashi, Chuo, Yamanashi Japan; 4 Antony- Xavier Interdisciplinary Scholastics (AXIS), GN Corporation Co. Ltd., Kofu, Yamanashi Japan; 5 Mary-Yoshio Translational Hexagon (MYTH), Nichi-In Centre for Regenerative Medicine (NCRM), Chennai, India; 6 R & D, Sophy Inc., Kochi, Japan; 7 Levy-Jurgen Transdisciplinary Exploratory (LJTE), Global Niche Corp, Wilmington, DE USA

**Keywords:** Medical research, Translational research

## Abstract

Recent advances in the management of Duchenne muscular dystrophy (DMD), such as exon skipping and gene therapy, though have reached a clinical stage, the outcome at its best is still considered suboptimal. In this study, we evaluated a novel N-163 strain of *Aureobasidium pullulans* produced β-glucan (Neu-REFIX) for its potential as an adjuvant to slow down the progression of the disease by anti-inflammatory and anti-fibrotic effects. In this study, 45 mice in the three groups, 15 each in a group; Gr. 1 normal mice, Gr.2 mdx mice as vehicle, and Gr.3 mdx mice administered the N-163 β-glucan for 45 days. The N-163 β-glucan group showed a significant decrease in the plasma ALT, AST, and LDH levels (126 ± 69 U/l, 634 ± 371 U/l, 3335 ± 1258 U/l) compared with the vehicle group (177 ± 27 U/l, 912 ± 126 U/l, 4186 ± 398 U/l). Plasma TGF-β levels increased, and plasma IL-13 levels decreased in the N-163 group. The inflammation score of HE-stained muscle sections in the N-163 group (1.5 ± 0.8) was lower than that in the vehicle group (2.0 ± 0.8). The N-163 strain β-glucan group (24.22 ± 4.80) showed a significant decrease in the fibrosis area (Masson’s Trichrome-positive area) compared with the vehicle group (36.78 ± 5.74). The percentage of centrally nucleated fibres evaluated by Masson’s trichrome staining was 0 in the normal group, while it increased to 80% in the vehicle group but remained at 76.8% in the N-163 group. The N-163 β-glucan group showed a significant decrease in the fibrosis area. Considering their safety and easy oral consumption, Neu-REFIX β-glucan could be worth large multicentre clinical studies as adjuvant in slowing down the progress of DMD.

## Introduction

Duchenne muscular dystrophy (DMD) is a devastating, severe, and progressive X-linked disorder in which mutations in the dystrophin gene lead to loss of functional dystrophin protein, making the muscle fibres prone to damage after skeletal muscle contraction. Muscle fibres tend to regenerate, but the process is not normal or complete. This continued cycle of damage and incomplete regeneration damages the muscle, and finally, the regenerative capacity of the fibres is exhausted, which leads to necrosis and is then replaced by adipose and connective tissue, leading to fibrosis. Prolonged activation of the innate immune response in DMD also leads to excessive chronic inflammation and additional tissue damage^1,2^.

The incidence of DMD is one per 5136 male births^3^. Symptoms of DMD are noticed around 3 years of age, and many patients become wheelchair-bound by 8–10 years of age. The life span is approximately 20 years, when cardiorespiratory failure results in death. Currently, there is no definitive cure for this condition. Therapeutic strategies are of two major types. The first approach is aimed at restoring the function of dystrophin which includes experimental approaches such as exon skipping, gene therapy, myostatin inhibitors, utrophin modulation, CRISPR/Cas9, and suppression of stop codons and stem cells. These dystrophin-targeted therapies have not yielded the intended outcome because they can slow down the progression but do not restore the function of abnormal muscle tissues due to the degenerative nature of DMD. In addition, it is difficult for these therapies to target all the muscle tissues that are widely distributed throughout the body. The second therapeutic approach targets pathological pathways, including fibrosis, inflammation, loss of calcium homeostasis, oxidative stress, ischaemia, and muscle atrophy. These adjunct therapies are gaining more attention because they offer relief against at least some of the symptoms in these patients^1^.

The X-linked muscular dystrophy (mdx) mouse is a widely used animal model for Duchenne muscular dystrophy (DMD), as it also develops an X-linked recessive inflammatory myopathy, and a deficit in the gene coding for dystrophin occurs in both. In mdx mice, the disease course is more benign, and the abrupt onset of muscle fibre degeneration with intense inflammatory infiltrates since weaning, with extensive myonecrosis occurring near adulthood accompanied by persistent fibrosis, followed by muscle regeneration, makes it a valuable animal model for studying inflammation, fibrosis, and muscle regeneration in DMD^4^.

Biological response modifier β-glucans (BRMGs) produced by N-163 strain of *Aureobasidium pullulans* (*A.pullulans*) are capable of eliciting anti-inflammatory and immune-modulating responses apart from metabolic regulation, proven in pre-clinical animal models^5,6^, and non-alcoholic steatohepatitis (NASH) model of STAM for their anti-fibrotic effect as well^7^. Followed by that, in a human clinical study, the N-163 strain produced BRMG has shown in 27 patients with DMD, significant anti-inflammatory effects evident from decrease of IL-6 and anti-fibrotic effects evident from decrease in TGF-β levels. Plasma dystrophin levels increased by up to 32% in that study and medical research council (MRC) grading showed muscle strength improvement in 12 out of 18 patients (67%) in the treatment group^8^. However, studying some of the intricate effects on inflammation and fibrosis by extensive muscle biopsies that are required is not feasible in human clinical studies.

Therefore, we performed the present study in mdx mouse model of DMD to study the effects of the N-163 strain produced BRMG.

## Methods

This study was conducted in accordance with the Animal Research: Reporting of In Vivo Experiments Guidelines. C57BL/10SnSlc mice (3 weeks old, male) were obtained from Japan SLC, Inc. (Japan). C57BL/10-mdx/Jcl mice (3 weeks old, male) were obtained from CLEA Japan (Japan). All animals used in this study were cared for in accordance with the Act on Welfare and Management of Animals (Ministry of the Environment, Act No. 105 of 1 October 1973), Standards Relating to the Care and Management of Laboratory Animals and Relief of Pain (Notice No.88 of the Ministry of the Environment, April 28, 2006) and the Guidelines for Proper Conduct of Animal Experiments (Science Council of Japan, 1 June 2006).

### Study groups

#### Group 1: Normal

Fifteen C57BL/10SnSlc mice were without any treatment until sacrifice.

#### Group 2: Vehicle

Fifteen mdx mice were orally administered vehicle [pure water] at a volume of 10 mL/kg once daily from days 0 to 45.

#### Group 3: N-163 β-glucan

Fifteen mdx mice were orally administered a vehicle supplemented with N-163 strain and produced β-glucan at a dose of 3 mg/kg as API in a volume of 10 mL/kg once daily from days 0 to 45.

The N-163 strain produced β-glucan (Neu-REFIX™), was provided by GN Corporation Co. Ltd. N-163 β-glucan was mixed with the required amount of RO water and stirred until it was completely dissolved. The solution was dispensed into seven tubes and stored at 4 °C until the day of administration. The dosing formulations were stirred before administration. The dosing formulations were administered within seven days.

- The vehicle and N-163 β-glucan, were administered orally at a volume of 10 mL/kg.
- The N-163 β-glucan was administered at a dose of 3 mg/kg API once daily.

The animals were maintained in an SPF facility under controlled conditions of temperature (23 ± 3 °C), humidity (50 ± 20%), lighting (12-h artificial light and dark cycles; light from 8:00 to 20:00), and air exchange. The animals were housed in TPX cages (CLEA Japan), with a maximum of five mice per cage.

Sterilised Paper-Clean (Japan SLC) was used for bedding and was replaced once a week. A sterilised normal diet was provided ad libitum and placed in a metal lid on top of the cage. RO water was provided ad libitum from a water bottle equipped with a rubber stopper and sipper tube. The water bottles were replaced once a week, cleaned, sterilised in an autoclave, and reused. Mice were identified using an ear punch. Each cage was labelled with a specific identification code. The mdx model mice were randomised into two groups of 15 mice based on their body weight, the day before the start of treatment. Randomisation was performed by body weight-stratified random sampling using Excel software. mdx model mice were stratified by body weight to obtain SD and the difference in the mean weights among groups was as small as possible.

Viability, clinical signs (lethargy, twitching, and laboured breathing), and behaviour were monitored daily. Body weight was recorded daily before treatment. The mice were observed for significant clinical signs of toxicity, morbidity, and mortality before and after administration. The animals were sacrificed on day 45 by exsanguination through the abdominal vena cava under isoflurane anaesthesia (Pfizer Inc.).

At study termination, urine samples were collected (> 50 µL) and stored at − 80 °C for biochemistry. At study termination, non-fasting blood was collected through the abdominal vena cava using precooled syringes. The collected blood was transferred to pre-cooled polypropylene tubes containing anticoagulants (Novo-Heparin) and stored on ice until centrifugation. The blood samples were centrifuged at 1000×g for 15 min at 4 °C. The supernatant was collected and stored at − 80 °C for biochemistry.

After sacrifice, the quadriceps, gastrocnemius, soleus, plantaris, tibialis anterior, extensor digitorum longus, diaphragm, and myocardium muscles were collected. Individual muscle weights were measured. Each muscle was separated, dissected, and stored, as described below.

1. The tragacanth gum was placed on cork disk, with only enough tragacanth gum to provide foundation for the oriented muscle.
2. The tragacanth gum on one end was placed so that the long axis of the muscle is perpendicular to the cork disc.
3. The specimen was rapidly frozen and placed into isopentane cooled in liquid nitrogen.
4. The frozen block was transferred on to dry ice and the isopentane was evaporated for around one hour,
5. The frozen block was stored at -80 °C.

### Measurement of plasma biochemistry

#### For all mice

Plasma ALT, AST, and LDH levels were measured by FUJI DRI-CHEM 7000 (Fujifilm Corporation).

#### For Sub-gr. A (n = 5 from each group)

Plasma cystatin C, and TGF-β levels were measured using commercial enzyme-linked immunosorbent assay (ELISA) kits.

#### For Sub-gr. B (n = 5 from each group)

Plasma IL-13 and haptoglobin levels were measured using commercial ELISA kits.

### Measurement of urine biochemistry

#### For all mice

Urine myoglobin and titin levels were measured by commercial ELISA kits.

ELISA kits are shown in Supplementary Table 1.

### Histological analyses

Sections were cut from paraffin blocks of the muscle (TBD) tissue using a rotary microtome (Leica Microsystems). After sectioning, each slide was coded as a number for blind evaluation. Each number was generated using the RAND function of the Excel software, sorted in ascending order, and assigned to the slides. The tissue slides were used for staining and evaluated by an experimenter.

For HE staining, sections were cut from frozen blocks of muscle (tibialis anterior) tissue and stained with Lillie-Mayer’s haematoxylin (Muto Pure Chemicals Co., Ltd., Japan) and eosin solution (FUJIFILM Wako Pure Chemical Corporation). The inflammation score was calculated according to the criteria of Tinsley^9^, as shown below:

### Inflammation score

0 = none to minimal—No inflammation within the muscle bundles or inter-bundle connective tissue; occasional mononuclear inflammatory cells may be present but no obvious aggregations.

1 = mild—Occasional mononuclear inflammatory cells in the inter-bundle connective tissue with focal aggregations of mononuclear inflammatory cells.

2 = moderate—Multiple foci of mononuclear inflammatory cell infiltration in the inter-bundle connective tissue; occasional mononuclear inflammatory cells between individual muscle fibres.

3 = severe—Multiple large foci of mononuclear inflammatory cell infiltration in the inter-bundle connective tissue extending into the intra-bundle connective tissue with expansion of the inter-bundle and intra-bundle spaces.

For Masson’s trichrome staining, frozen muscle (diaphragm, 7 mice/group) sections were stained in Weigert’s iron haematoxylin working solution (Sigma-Aldrich), Biebrich Scarlet-Acid fuchsin solution (Sigma-Aldrich), Phosphotungstic/phosphomolybdic acid solution, aniline blue solution, and 1% acetic acid solution (Sigma-Aldrich).

For quantitative analysis of the fibrotic area, bright field images of Masson’s trichrome-stained sections were captured using a digital camera (DFC295; Leica, Germany) at 200- fold magnification and the positive areas in five fields/section (TBD) were measured using ImageJ software (National Institute of Health, USA).

### Statistical tests

Statistical analyses were performed using the Prism Software 6 (GraphPad Software, USA). Statistical analyses were performed using the Bonferroni’s multiple comparison test. Comparisons were made between the following groups: (1) Group 2 (Vehicle) versus Group 1 (Normal) and Group 3 (N-163 β-glucan). Statistical significance was set at *P* < 0.05. Results are expressed as mean ± SD. A trend or tendency was assumed when a one-sided t-test returned *P*-values of < 0.1. Comparisons were made between the following groups.

1. Group 2 (Vehicle) vs. Group 1 (Normal)
2. Group 2 (Vehicle) vs. Group 3 (N-163 β-glucan)

### Ethics approval

Protocol approvals were obtained from SMC Laboratories, Japan’s IACUC (Study Protocol no: SP_SLMA143-2208-3). This study was conducted in accordance with the Animal Research: Reporting of In Vivo Experiments Guidelines. C57BL/10SnSlc mice (3 weeks of age, male) were obtained from Japan SLC, Inc. (Japan). C57BL/10-mdx/Jcl mice (3 weeks of age, male) were obtained from CLEA Japan (Japan). All animals used in this study were cared for following guidelines: Act on Welfare and Management of Animals (Ministry of the Environment, Act No. 105 of October 1, 1973), Standards Relating to the Care and Management of Laboratory Animals and Relief of Pain (Notice No.88 of the Ministry of the Environment, April 28, 2006) and Guidelines for Proper Conduct of Animal Experiments (Science Council of Japan, June 1, 2006).

## Results

### Body weight changes were not significant (Fig. 1)

**Figure 1:**
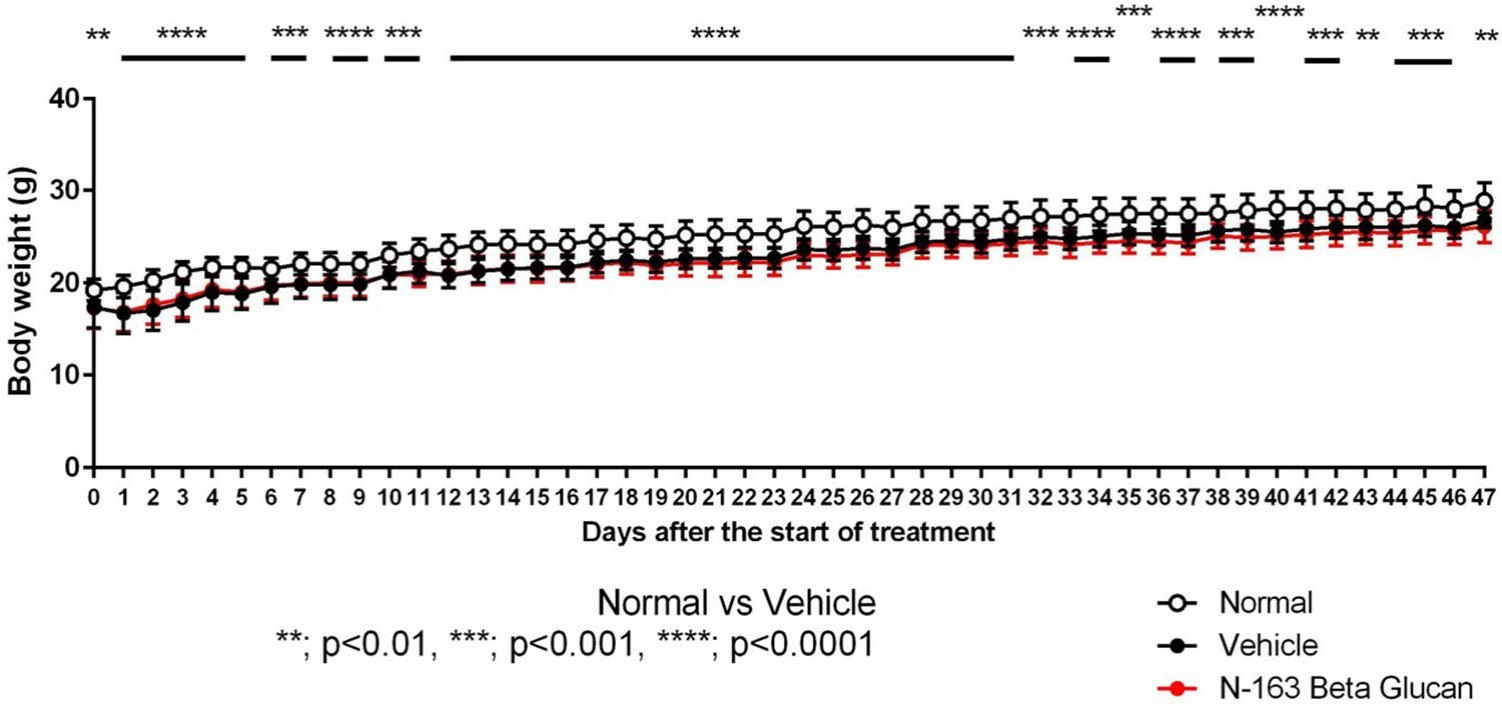
Changes in body weight.

The mean body weight of mice in the vehicle group was significantly lower than that of mice in the normal group during the study period. There was no significant difference in the mean body weight between the vehicle group and the N-163 β-glucan.

### N-163 strain β-glucan almost normalised the levels of liver enzymes in mdx mice

**Figure 2:**
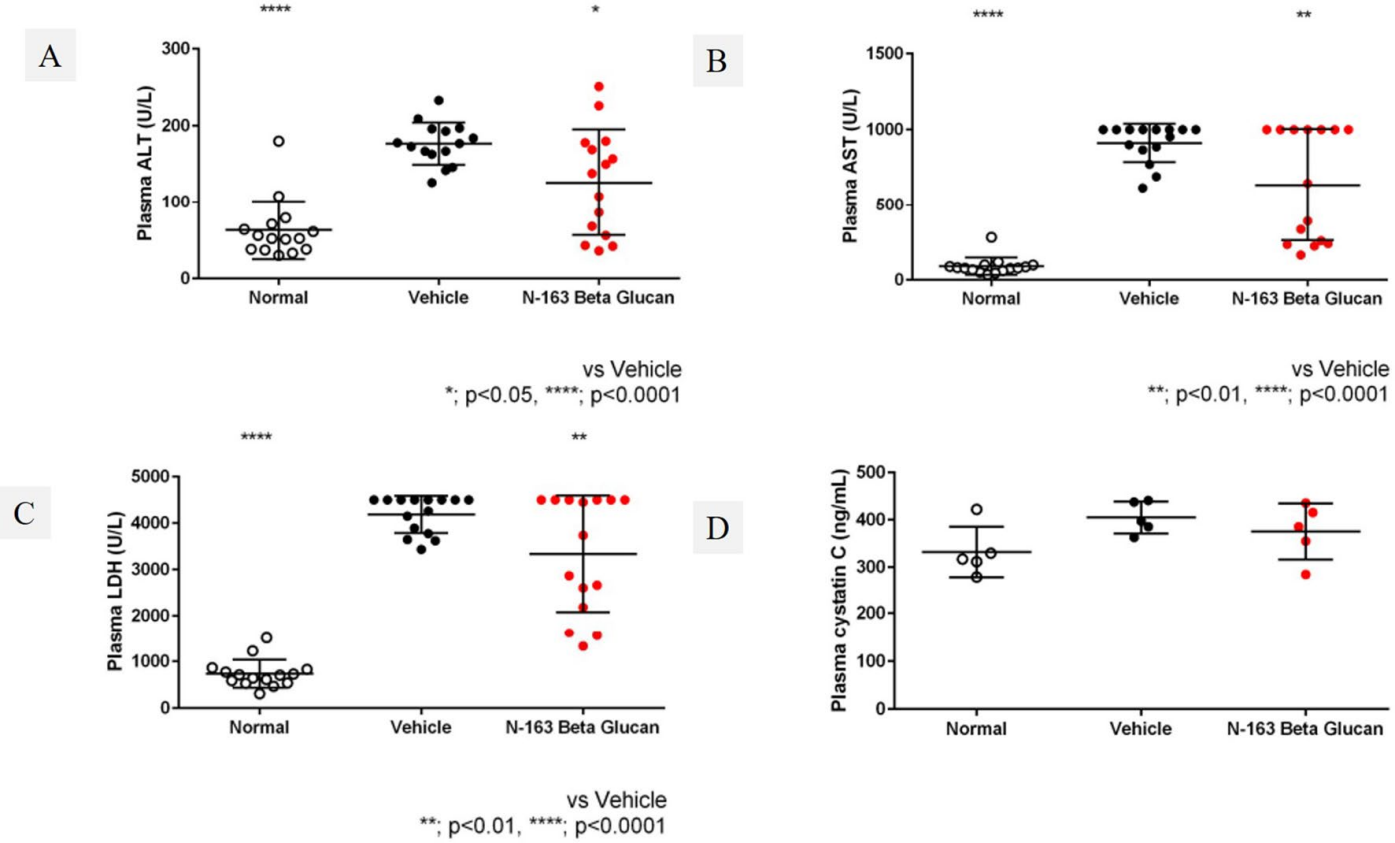
Changes in plasma ALT, AST, LDH, and cystatin levels.

**Figure 3:**
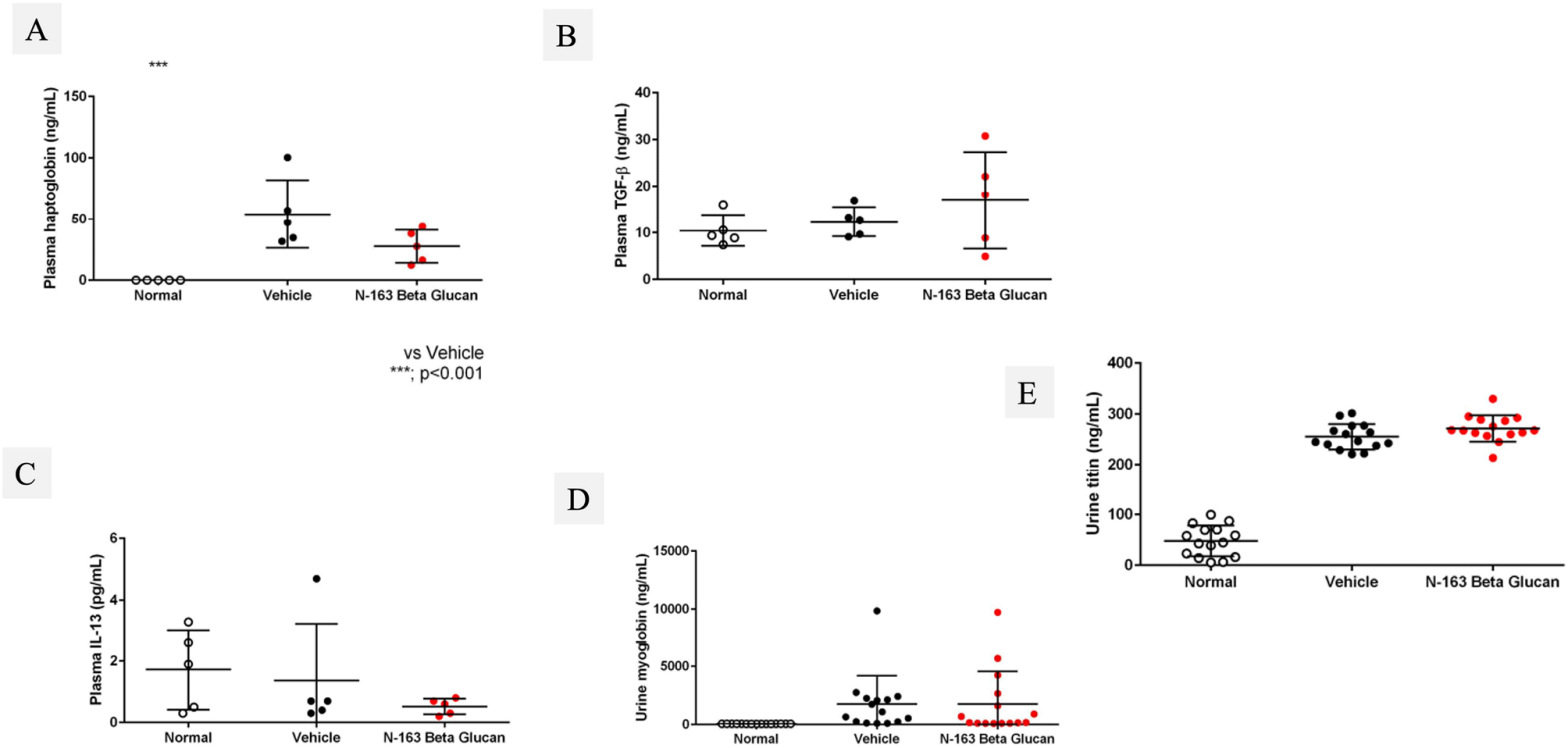
Changes in plasma haptoglobin, TGF-β, IL-13, and Urine myoglobin, titin levels.

The vehicle group showed a significant increase in plasma ALT (177 ± 27 U/l), AST (912 ± 126 U/l), and LDH (4186 ± 398 U/l) levels compared to the normal group (63 ± 38, 90 ± 58, 737 ± 298 U/l) (Fig. 3). The N-163 β-glucan group showed a significant decrease in the plasma ALT, AST, and LDH levels (126 ± 69, 634 ± 371, 3335 ± 1258 U/l) compared with the vehicle group. Plasma cystatin C level in the Vehicle group (405.1 ± 33.7 ng/ml) tended to increase compared with the normal group (332.1 ± 53.7 ng/ml). There was no significant difference in plasma cystatin C levels between the vehicle group and the N-163 β-glucan group (375.3 ± 59.1 ng/ml) (Fig. 2). The vehicle group showed a significant increase in the plasma haptoglobin level (54.2 ± 27.8 ng/ml) compared with the Normal group (0.0 ± 0.0 ng/ml). Plasma haptoglobin levels in the vehicle group tended to decrease compared with the N-163 strain β-glucan group (27.6 ± 13.5 ng/ml).

### N-163 β-glucan reduced inflammation in the serum and muscle of mdx mice

**Figure 4:**
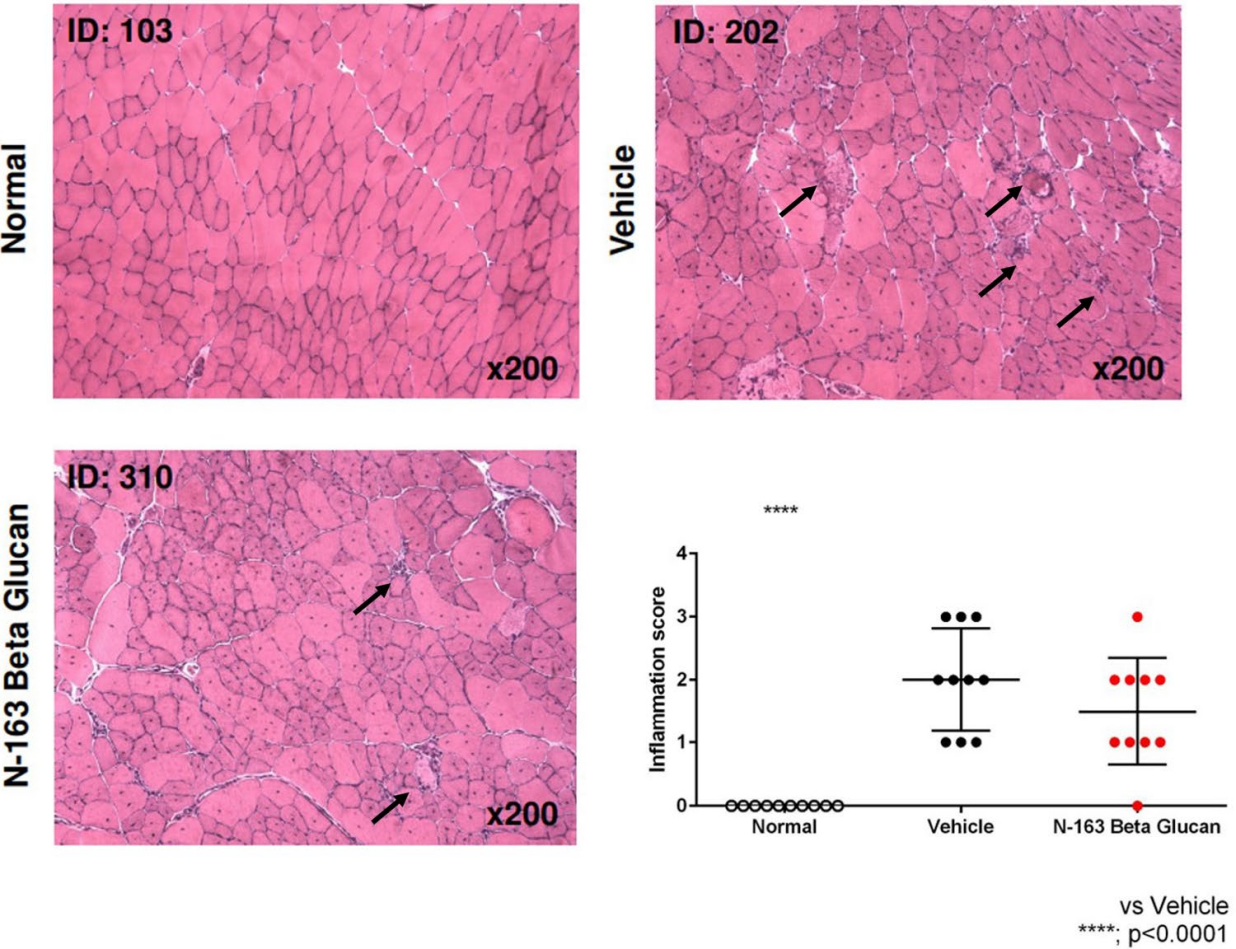
Representative photomicrographs of HE-stained muscle sections (**A**—Normal; **B**—Vehicle; **C**—N-163 strain produced β-glucan); arrows indicate foci of inflammation and (**D**) inflammatory score as per Tinsley et al.^9^; Scale bar = 20 µm.

Plasma TGF-β levels increased, and plasma IL-13 levels decreased in the N-163 group (Fig. 3). Urine myoglobin level in the Vehicle group (1758.0 ± 2440.0 ng/ml) tended to increase compared with the Normal group (63.0 ± 2.4 ng/ml). There was no significant difference in urine myoglobin levels between the Vehicle group and the N-163 strain β-glucan group (1774.0 ± 2792.0 ng/ml). The Vehicle group showed a significant increase in urine titin level compared with the normal group (47.9 ± 30.5 ng/ml). Urine titin levels in the Vehicle group (255.4 ± 25.2 ng/ml) tended to be lower than that in the N-163 β-glucan group (271.9 ± 26.1 ng/ml) (Fig. 3E). Representative photomicrographs of haematoxylin and eosin (HE)-stained muscle sections are shown in Fig. 4. The Vehicle group showed a significant increase in the inflammation score (2.0 ± 0.8) compared with the normal group (0.0 ± 0.0). The inflammation score in the N-163 β-glucan group (1.5 ± 0.8) tended to decrease compared with the vehicle group.

## Masson’s trichrome staining

### N-163 β-beta glucan significantly reduced the fibrosis area in mdx mice

**Figure 5:**
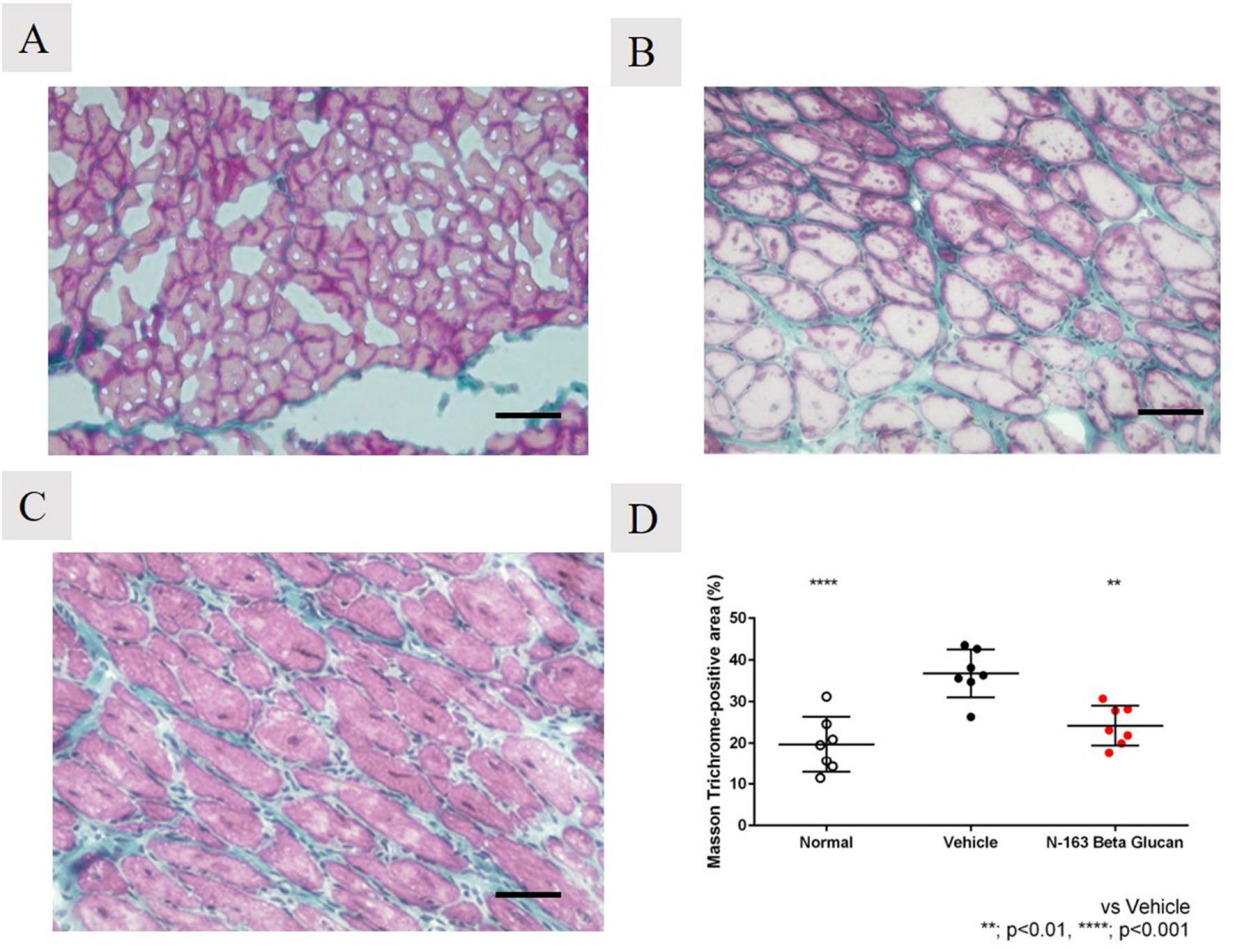
Representative photomicrographs of Masson’s Trichrome-stained muscle sections (**A**—Normal; **B**—Vehicle; **C**—N-163 strain produced β-glucan) and Fibrosis area (Masson’s Trichrome positive area); Scale bar = 20 µm.

**Figure 6:**
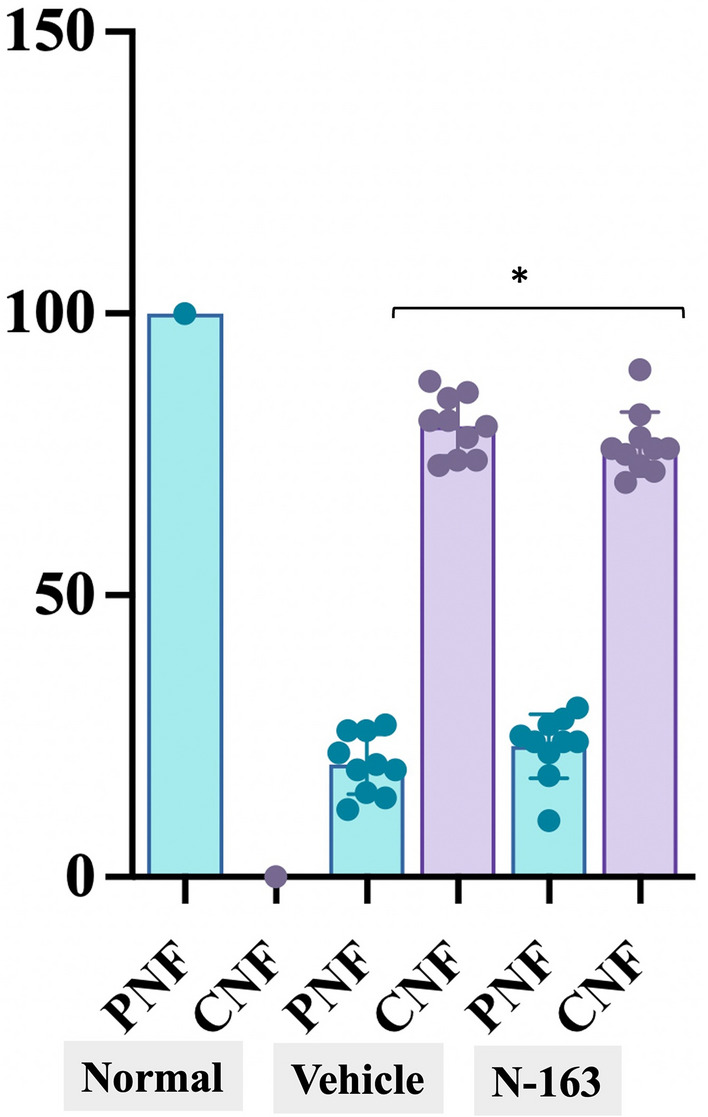
Percentage of peripherally nucleated fibres (PNF) and centrally nucleated fibres (CNF) in the three groups, Normal mice (Normal), mdx mice (Vehicle), mdx mice administered N-163 beta-glucan (N-163).

In Masson’s trichrome staining, the vehicle group (36.78 ± 5.74) showed a significant increase in the fibrosis area (Masson’s trichrome-positive area) compared with the normal group (19.68 ± 6.73) (Fig. 5). The N-163 strain β-glucan group (24.22 ± 4.80) showed a significant decrease in the fibrosis area (Masson’s Trichrome-positive area) compared with the vehicle group. (Fig. 5). The percentage of centrally nucleated fibres (CNF) was 0 in the normal group, 80 ± 5.292% in the vehicle group, and 76.8 ± 5.692% in the N-163 group (*p*-value = 0.20) (Fig. 6).

## Discussion

The objective of this study was to slow down the progression of DMD pathology to ensure a longer lifespan. According to literature, the life span of patients diagnosed with DMD in the 1960s was 14.4 years^10^ which gradually increased to 25.3 years in the 1990s through 2000. Recently, it has been reported to be 39.6 years^11^, which has not been attributed to definitive treatment but to the disease-modifying and anti-inflammatory treatment approaches. Having accomplished an average increase of life span from 14.4 to 39.6 years in four decades with such supporting approaches, this study was also aimed to evaluate the efficacy of the BRMGs in slowing down the disease progress. However, the advantages of the N-163 BRMG considered were their ease of oral consumption, allergen-free, and adverse reaction-free track record as a food supplement against the adverse reactions of the other supporting treatments, such as excessive weight gain, increased risk of bone fractures due to bone density reduction, development of cataracts, and increased intraocular pressure^12^.

In the earlier clinical study plasma biomarkers showed significantly beneficial changes. Inflammatory markers such as ALT and AST levels tend to be elevated in mdx mice because of leakage from the diseased muscle tissues. In the current study, the decrease in AST and ALT levels was higher in the N-163 group than in the vehicle and control groups^13^. With increased LDH levels reported in the literature for mdx mice^14^, the current study showed that LDH values decreased after N-163 treatment. Increased lipopolysaccharides lead to chronic inflammation, and treatment with adiponectin (ApN) has been able to provide therapeutic benefits in DMD through insulin-sensitising, fat-burning, anti-inflammatory, and anti-oxidative stress properties. In the present study, supplementation with N-163 BRMG has been able to decrease LDH levels which may be helpful in alleviating chronic inflammation^15^. Dramatic elevation in urinary titin excretion has been reported in patients with DMD and dystrophin-deficient rodents, coinciding with the development of systemic skeletal muscle damage^16^. In this study, there was a significant decrease in urine titin levels after N-163 BRMG administration, indicating the resolution of skeletal muscle damage. The inducible plasma marker haptoglobin is an acute phase response protein secreted in relation to tissue damage and sterile inflammation, and has been reported to be elevated in DMD mice^14^. In the current study, a significant decrease in the plasma marker haptoglobin was observed after N-163 administration. Although TGF-β has been reported to be involved in fibrosis, studies have shown that TGF-β also functions as an anti-inflammatory cytokine and helps balance inflammation and fibrosis^17^. Treatment with steroids and vitamin in a study has been shown to decrease IL-13, a pro-fibrotic marker^18^. In the current study, N-163 BRMG treatment decreased IL-13 levels. In the present study, TGF-β levels were increased in the N-163 group. The N-163 β-glucan showed a significant decrease in the fibrosis area (Masson’s trichrome-positive area) compared with the vehicle group. The inflammation score and fibrosis area in the N-163 β-glucan group tended to decrease compared with the vehicle group. In DMD, sporadic dystrophin-positive muscle fibres, called revertant fibres (RFs), are thought to arise from skeletal muscle precursor cells and clonally expand with age owing to the frequent regeneration of necrotic fibres^19^. The nuclei of newly regenerated muscle fibres are centrally located, whereas those of mature muscle fibres are peripherally located. In our study, CNF increased in mdx mice compared to that in normal mice due to an increase in necrotic fibres. The number of CNF showed decrease after N-163 β-glucan compared to the vehicle group, though not significant, we speculate that it could be due to necrosis being tackled by N-163 β-glucan which we infer by reduction in inflammation score, and also perhaps, the number of peripheral nucleated fibres having increased after N-163 β-glucan, showing that normal fibres that are matured are also increased after N-163 administration. However, these observations need further validation in longer duration studies.

The N-163 strain of *A.pullulans* produced BRMG is a food supplement whose safety and efficacy have been proven in several studies^5–7,20,21^. It is manufactured in a GMP facility. The N-163 β-glucan food supplement has been reported to have beneficial immunomodulatory effects, anti-inflammatory and anti-fibrotic effects apart from beneficial reconstitution of the gut microbiome in pre-clinical and clinical studies of metabolic diseases, Non-alcoholic Steatohepatitis (NASH) and COVID-19^5,6,20,22^. Having proven that the N-163 β-glucan hence can be safely orally consumed and that it has both systemic anti-inflammatory efficiency with significant effects on beneficially manipulating organ fibrosis, in the current study, we have studied its effects only on inflammatory and fibrosis parameters of skeletal muscles beside plasma biomarkers, and they have been found to be beneficially modulated, further demonstrating the potential of this N-163 strain produced β-glucan. The effects on dystrophin, both skeletal and smooth muscle produced, must be evaluated because in the human study of DMD, it is to be noted that plasma dystrophin levels increased by up to 32%^8^. Furthermore, strong emerging evidence has reported that the primary cause of DMD is the lack of dystrophin in the smooth muscle of blood vessels rather than in the skeletal or cardiac muscle^23^ and hence, additional evaluations of cellular and molecular changes in the vascular system of this pre-clinical model may have to be undertaken to prove the significance of the N-163 β-glucan. Apart from the evaluation of dystrophin levels, other functional parameters at the muscular genetic and epigenetic levels need to be studied as well. The disease-modifying approach such as the N-163 strain β-glucan, we have reported is of more significance to those patients above the age of 18 years, in whom dystrophin producing satellite stem cells would have got depleted or dysfunctional^24^ and therefore they may not benefit from the gene therapy or exon skipping therapies. Having also proven with safety and efficacy in the earlier human clinical study in patients younger than 18 years when consumed along with conventional treatments, they are also worth recommending at any disease stage, across age groups as an adjuvant in the management of DMD.

This study in mdx mice, having evaluated only the skeletal muscle fibrosis and inflammation, has certain limitations; i. there are different models of DMD mice such as dystrophin-utrophin double knockout (dko) mice^25^ and the effects of the N-163 strain produced β-glucan needs to be studied in other such mice and animal models of DMD; ii. Effects only on skeletal muscle has been studied, evaluation of effects in cardiac muscle and vascular smooth muscle^26^ must be studied; iii. The study was performed only for a short duration of 45 days and longer duration studies are needed for validation.

## Conclusion

Dietary supplementation with the N-163 strain of *A. pullulans* produced BRMG (Neu-REFIX) has proven to be safe and was found to ameliorate inflammation in mdx mice, proven by a significant decrease in inflammation score and fibrosis, levels of plasma ALT, AST, LDH, IL-13, and haptoglobin, with an increase in anti-inflammatory TGF-β, apart from balanced regulation of the quantity of CNF fibres in this study of 45 days duration. Having previously proven its potential in human clinical studies confirming the safety and efficacy of plasma-based biomarkers, we recommend that this Neu-REFIX β-glucan be validated in long-term multi-centric clinical studies for its potential as a disease-modifying adjuvant in slowing the progress and increasing the life span of patients with DMD at any stage of the disease.

### Supplementary Information

Supplementary Table 1.

## Data Availability

All data generated or analysed during this study are included in the article itself.
