# Supplementary material for: Resolution of fibrosis in mdx dystrophic mouse after oral consumption of N-163 strain of Aureobasidium pullulans produced β-glucan
Source: Sci Rep. 2023 Oct 9;13:17008. doi: 10.1038/s41598-023-44330-0 (PMC10562469; doi:10.1038/s41598-023-44330-0)
Supplement: Supplementary file 1 — Supplementary Table 1. [file 41598_2023_44330_MOESM1_ESM.docx]

**Table 1. ELISA kit list**

| Protein | Details |
| --- | --- |
| Cystatin C | Name: Mouse/Rat Cystatin C Quantikine ELISA Kit Manufacturer: R&D Systems  Lot #: P321999 Cat #: MSCTC0 |
| TGF-β | Name: TGF-β, Canine/Mouse/Porcine/Rat, ELISA Kit, Quantikine, 2^nd^ Generation  Manufacturer: R&D Systems Lot #: P324187  Cat #: MB100B |
| IL-13 | Name: Mouse IL-13 ELISA Kit Manufacturer: Abcam  Lot #: GR3240324-1  Cat #: ab219634 |
| Haptoglobin | Name: Mouse Haptoglobin ELISA Kit Manufacturer: Abcam  Lot #: GR3410593-1  Cat #: ab157714 |
| Myoglobin | Name: MOUSE MYOGLOBIN ELISA  Manufacturer: Life Diagnostics Lot #: MYO1E0622  Cat #: MYO-1 |
| Titin | Name: Mouse Titin N-Fragment Assay Kit Manufacturer: IBL  Lot #: 2D-222 Cat #: 27602 |
